# Effect of vertical distance between the heart base and the cuff on arterial blood pressure measurements using PetMAP in anaesthetised dogs

**DOI:** 10.1111/jsap.70148

**Published:** 2026-06-04

**Authors:** R. McConomy, H. Pinnok, C. Adami

**Affiliations:** ^1^ Department of Veterinary Medicine University of Cambridge Cambridge UK

## Abstract

**Objectives:**

To evaluate how variations in limb positioning, when compared with standard position at the heart base, affect oscillometric arterial blood pressure measurements in 22 anaesthetised healthy dogs.

**Materials and Methods:**

The thoracic vertical depth was measured in each dog from the caudal angle of the scapula to the body of the sternum caudal to the olecranon, and 20% of this length was used as deviation from the heart base level, identified by the humerus greater tubercle, for the arterial blood pressure measurement above and below the heart base. Measurements were taken in the thoracic and pelvic limbs with the cuff aligned with the heart base, and with the cuff positioned above and below the heart base by the deviation, always with the dogs in dorsal recumbency.

**Results:**

Both systolic (*P* = .02) and mean arterial blood pressure (*P* = .047) values were significantly increased when the thoracic limb was lowered compared to heart base. The absolute difference in mean arterial blood pressure between heart base and values measured below the heart base was 3.7 (3.0 to 8.3) mmHg and positively correlated with the dogs' body weight (*P* < .001). For systolic (*P* = .030), mean (*P* = .015) and diastolic arterial blood pressure (*P* = .025), values were significantly decreased when the pelvic limb was elevated compared to heart base. The absolute difference in mean arterial blood pressure between heart base and values above the heart base was 6.1 (1.6 to 10.6) mmHg.

**Clinical Significance:**

Deviations as little as 3 cm above or below the heart base resulted in mean arterial blood pressure misreadings of up to 6 mmHg, representing a clinically relevant discrepancy.

## INTRODUCTION

In order to ensure adequate tissue perfusion, oxygen delivery and removal of metabolic waste products during anaesthesia, monitoring of arterial blood pressure (ABP) has been listed between the “minimum recommendations” by the Small Animal Anesthesia and Sedation Monitoring Guidelines developed by the American College of Veterinary Anesthesia and Analgesia (Bailey et al., 2025).

Ideally, the gold standard method to measure ABP is invasively through arterial catheterisation. However, since this method is unpractical and not without risk, for routine procedures non‐invasive methods are preferentially used instead (Acierno et al., 2018; Bailey et al., 2025). When monitoring of ABP relies on oscillometry, it is recommended that measurements are taken at least every 5 minutes.

Both Doppler ultrasonic flow detector technology and oscillometry are widely used options for non‐invasive ABP measurement in small animals, with many oscillometry devices now available on the veterinary market (Armour et al., 2024; Mantovani et al., 2025). Among them, PetMAP devices became popular during the last decade and were found non‐inferior to Doppler in anaesthetised dogs (Lyberg et al., 2021; Rattez et al., 2010; Vachon et al., 2014). One study found that PetMAP had better agreement with invasive ABP measurement than Doppler in anaesthetised dogs, although systolic pressure was slightly underestimated by both devices with a calculated bias for PetMAP of −6.1 mmHg (Vachon et al., 2014).

A consensus statement of the American College of Veterinary Internal Medicine established a protocol for accurate ABP measurement. Based on this, an appropriate cuff size (30% to 40% of circumference of the limb at the point where the cuff is going to be positioned) should be selected, and the vertical distance from the heart base to the measuring cuff should be as little as possible, ideally equal to 0 cm (Acierno et al., 2018). In dogs positioned in dorsal recumbency, the heart base, identified with the right atrium, is located at the same height as the greater tubercle of the humerus; this level is usually referred to as “standard cuff positioning” (Vet Visions, 2026).

Standard cuff positioning may be achievable in conscious dogs positioned in sternal recumbency; however, in anaesthetised dogs, body and limbs positioning on the operating table are likely to be dictated by the clinical procedure. Although a previous study in anaesthetised cats positioned in dorsal recumbency found that elevating the cuff 5 cm above heart base level resulted in clinically significant underestimation of ABP measured using PetMAP, the effect of raising or lowering the limb on which the cuff is placed during oscillometric ABP reading remains unknown in dogs (Franchino et al., 2024).

The aim of the present study was to evaluate how variations in limb positioning, when compared with the standard reference position, affect ABP values measured with PetMAP Graphic II in dogs under general anaesthesia.

## MATERIALS AND METHODS

### Study population

Dogs presented for elective neutering procedures at the Queen's Veterinary School Hospital between May 2024 and March 2025 were selected for this study. Approval was obtained by the Ethics and Welfare Committee of the Department of Veterinary Medicine at the University of Cambridge (Licence number: CR796; approval date: March 26, 2024). Signed written consent was obtained from dog owners prior to commencing data collection. Inclusion criteria were good health based on both physical examination and clinical history, and American Society of Anaesthesiologists (ASA) physical status classification of I out of V, as assigned by the anaesthesiologist in charge (de Almeida Lupiano et al., 2025). Dogs were excluded from the study if their temperament did not allow a complete physical examination, if they presented with any clinical condition resulting in ASA physical status classification higher than I, or if their limb circumference did not allow placement of an appropriately sized blood pressure cuff.

### Sample size calculation

The assumptions to calculate the sample size were based on previous research work conducted on cats, on pilot data collected during the phases of study design, and on a formula and derived equation (Franchino et al., 2024).

Based on the formula

$$\mathrm{\Delta bp}=d_{v}\cdot \frac{{\mathrm{sg}}_{b}}{{\mathrm{sg}}_{m}}$$

where bp is the measured ABP (mmHg), *d*_v_ is the vertical distance from the right atrium (in mm), sg_b_ is the specific gravity of blood (1.05 at 37°C) and sg_m_ is the specific gravity of mercury (Hg, ~13.6), it has been determined that, in human patients, ABP values change by approximately 0.77 mmHg for every cm of distance between the cuff and the level of the right atrium (Byrd & Brook, 2014).

In order to translate the applicability of this information from the human species to dogs, the following equation was derived from the above formula and used to calculate the sample size:

8.2cm:12cm=0.77mmHg:X

where 8.2 cm is the average diameter of the canine left ventricle in beagle dogs (Crippa et al., 1992), 12 cm is the average diameter of the human heart, 0.77 is the expected change in mmHg per cm of distance from the heart base and X (calculated as 1.12 mmHg) represents the estimated error per cm of distance between the level of the canine heart base and the cuff position.

Based on the above, assuming that a hypothetical 5 cm distance from the heart base would result in 1.12 × 5 = 5.6 (rounded to 6) mmHg difference in an average beagle dog, variables for sample size calculation were set as follows: mean A (MAP measured at heart base level): 70 mmHg with 10% SD, mean B: (MAP measured at 5 cm distance from heart base level): 76 mmHg (obtained by adding 6 to 70), alpha value: 0.05, enrolment ratio: 1:1 and power: 80%, our sample size calculation resulted in a minimum of 21 dogs in total, each to be used as its own control.

### Determination of the cuff‐heart base distance

The dorsoventral vertical depth of the thorax was measured with a measuring tape in each dog in standing posture before premedication, using a line of measurement from the caudal angle of the scapula to the body of the sternum, just caudal to the olecranon process (Fig 1). This variable was introduced to make the results comparable across different sizes and breeds of dogs. Considering that such depth corresponds to 20 to 22 cm in average sized beagle dog, and that 5 cm (the distance from the heart base previously used for cats) corresponds to approximately 20% of that depth value, in each dog for the ABP measurement above and below the heart base the cuff‐heart base distance was set as 20% of their thoracic vertical depth, defined in the current study as “deviation” (Franchino et al., 2024; IMAIOS Vet‐Anatomy Atlas, 2026).

**FIG 1:**
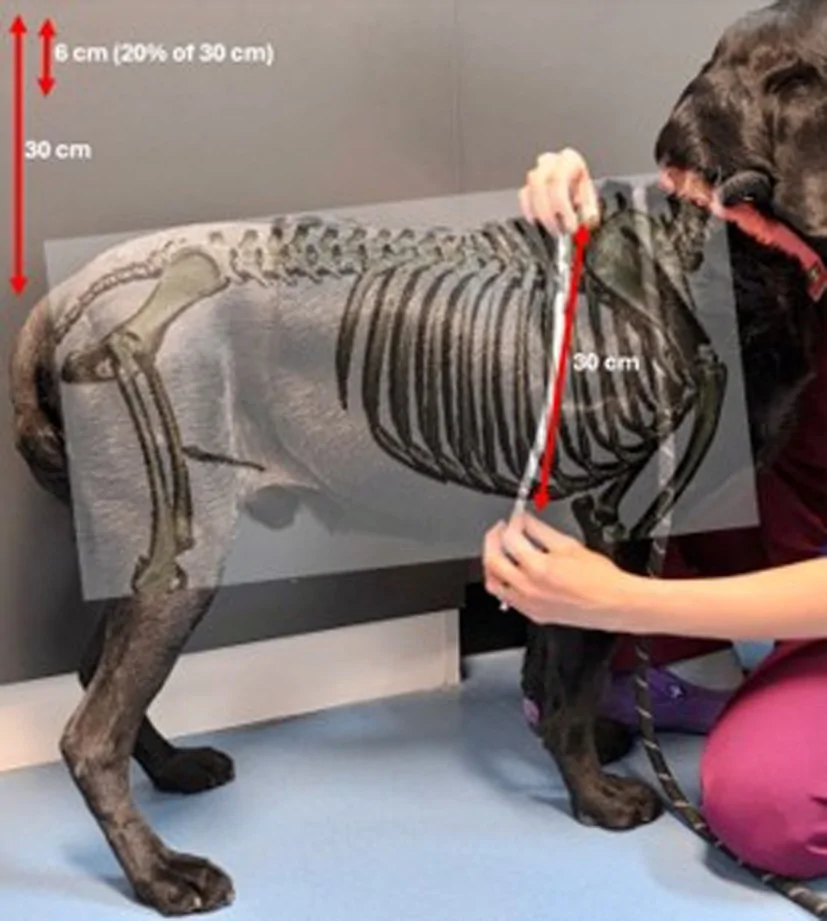
Measurement of the dorsoventral vertical depth of the thorax using a measuring tape in the conscious standing dogs, from the caudal angle of the scapula to the body of the sternum just caudal to the olecranon. The 20% of the measured value was calculated and used as each individual dog deviation, above and below the level of the base of the heart.

### Anaesthesia and ABP measurements

Dogs were admitted to the hospital 1 to 4 hours before the anaesthetic event. Food was withdrawn 8 to 12 hours prior to induction. All dogs were anaesthetised with a patient‐specific protocol, approved by the board‐certified anaesthesiologist in charge for the day, as per hospital policy.

During anaesthesia, peripheral arterial oxygen saturation, end‐tidal carbon dioxide, respiratory rate, end‐tidal isoflurane concentrations, electrocardiogram, heart rate and oesophageal temperature were measured using a multiparameter monitor and manually recorded every 5 minutes.

Measurements were initiated 15 minutes after the start of inhalational anaesthesia, to ensure that study data were obtained at a steady end‐tidal isoflurane concentration, once readings had remained consistent to one decimal place (0.1%) for at least 5 minutes. In order not to increase the duration of anaesthesia for reasons related to the study, the measurements occurred during clipping and disinfection of the surgical site. Arterial blood pressure was measured using the petMAP Graphic II device (Ramsey Medical, Inc., FL, USA), with the dogs positioned in dorsal recumbency. The same unit, which had been serviced by the seller at the start of the study period, was used throughout the clinical trial. All measurements were carried out by the same investigator.

An appropriately sized blood pressure cuff, with width between 40% and 45% of the limb circumference, was selected for both the antebrachium and pes. The cuff index line was placed within the “optimum zone” marked on the cuff, as per manufacturer instructions. The blood pressure cuff was applied so that it would be sufficiently tight to remain in place without causing tissue damage or impacting regional blood flow (Franchino et al., 2024). Measurements were taken using the optimised mode for canine thoracic and pelvic limb, respectively. To minimise systematic biases, the order in which measurements were taken for each limb and for each position relative to the heart base level (above or below) was determined using a randomisation sequence generated by a web‐based true random number generator service (random.org; Haahr, 2026).

Three measurements, for a total of 18 measurements per dog, were taken at 1‐minute intervals for each limb and position: with the centre of the blood pressure cuff aligned at the level of the heart base (along a horizontal line drawn through the greater tubercle of the humerus), with the cuff elevated above the heart base by the deviation and with the cuff lowered below the heart base by the deviation (Vet Visions, 2026). To ensure correct limb positioning, a loose tie was made around the carpus or tarsus with a broad open‐knitted bandage and then secured to a height adjustable stand.

### Data analysis

Demographic variables were analysed with descriptive statistics while data distribution was assessed for normality with the Shapiro–Wilk test. ABP values (SAP, MAP and DAP) measured at the level of the heart base were compared with both ABP values measured above and below the heart base, using either the paired *t*‐test or the Wilcoxon signed‐rank test, depending on data distribution.

Where values were significantly different to heart base values, absolute difference was calculated between the two values, and correlation between absolute differences and demographic variables (age and body weight of the dogs) was assessed with the Pearson product–moment correlation coefficient.

*P* < .05 was considered statistically significant. Commercially available software (IBM SPSS and SigmaStat version 4.0 and SigmaPlot version 10.0) was used for data analysis.

Data were presented as either means and standard deviations or medians and interquartile ranges, based on data distribution within each data set.

## RESULTS

### Demographics data and anaesthesia

A total of 22 dogs, of which 12 were entire females and 10 entire males, were included in this study. The dogs were aged 29 (18 to 37) months and their body weight was 11.5 kg (5.6 to 27.0 kg). Overall deviation from the level of the heart base was 3.1 (2.8 to 4.0 cm).

Dogs were premedicated either intramuscularly (im) or intravenously (iv) with a combination of either medetomidine (7.5 ± 2.5 μg/kg) or dexmedetomidine (5 ± 3 μg/kg) and methadone (0.2 ± 0.1 mg/kg), and general anaesthesia was induced with propofol titrated to effect (3 ± 1 mg/kg). Tracheal intubation with a suitable sized endotracheal tube was performed in all dogs, anaesthesia was maintained with isoflurane in 100% oxygen using either a Mapleson D or a circle breathing system. Intratesticular blocks and midline celiotomy incisional infiltration were performed on male and female dogs, respectively, using lidocaine (2 mg/kg). Postoperative analgesia consisted of subcutaneous carprofen (4 mg/kg) and, in the female dogs, either subcutaneous or intravenous buprenorphine (0.02 mg/kg).

### Measurements at the thoracic limb

Details of ABP values measured at the thoracic limb are presented in Table 1. For both SAP (*P* = .02) and MAP (*P* = .047), values were significantly increased when the limb was lowered with respect to heart base (Fig 2).

**Table 1:** Values of systolic (SAP), mean (MAP) and diastolic (DAP) arterial blood pressure measured in 22 anaesthetised dogs at the thoracic limb at the level of the heart base, and above and below the heart base

Measurements	Medians (ranges)	Means ± SD	Test	*P* value
**SAP (mmHg)**				
Heart base	105 (99 to 115)	110 ± 17		
Above heart base	103 (98 to 112)	106 ± 32	Wilcoxon signed ranked test^‡^	.100
Below heart base	114 (102 to 130)	113 ± 18	Paired *t*‐test^‡^	.020*
**MAP (mmHg)**				
Heart base	78 (71 to 81)	78 ± 10		
Above heart base	76 (70 to 83)	75 ± 33	Wilcoxon signed ranked test^‡^	.231
Below heart base	81 (71 to 91)	81 ± 10	Paired *t*‐test^‡^	.047*
**DAP (mmHg)**				
Heart base	61 (55 to 67)	62 ± 10		
Above heart base	58 (51 to 69)	55 ± 0.5	Wilcoxon signed ranked test^‡^	.177
Below heart base	64 (55 to 73)	64 ± 9	Paired *t*‐test^‡^	.095

^‡^ For comparison with baseline values

* *P* values are statistically significant

**FIG 2:**
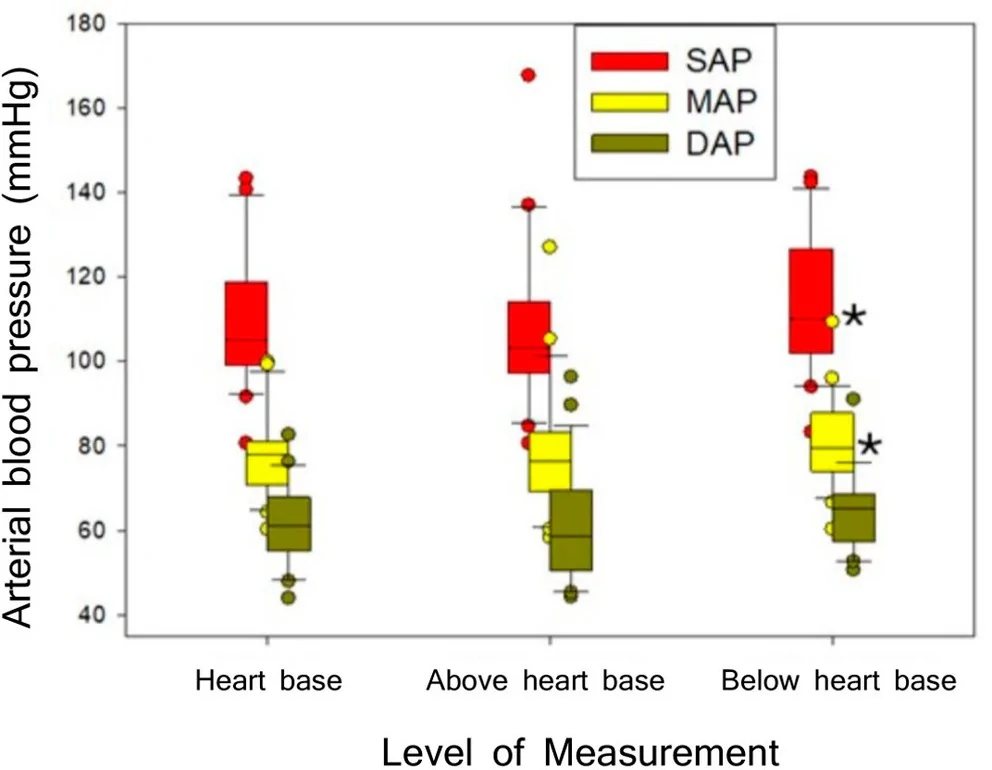
Values of systolic (SAP), mean (MAP) and diastolic (DAP) arterial blood pressures measured with PetMAP Graphic II at the thoracic limb of 22 anaesthetised dogs, with the cuff positioned at the level of the heart (baseline), and above and below the heart. The boxes represent the second and third quartiles, the horizontal line inside the boxes the medians, and the vertical lines either side of each box the lower (25%) and upper (75%) quartiles. The dots represent the outliers. The stars indicate statistical significance.

The absolute difference in SAP values measured between heart base and below was 2.8 (1.3 to 8.0) mmHg. No significant correlations were found between the magnitude of that difference and both the dogs' age (Pearson correlation coefficient: 0.18; *P* = .422) and their body weight (Pearson correlation coefficient: 0.24; *P* = .279).

The absolute difference in MAP values measured at heart base and below was 3.7 (3.0 to 8.3) mmHg. No significant correlations were found between the magnitude of that difference and the dogs' age (Pearson correlation coefficient: 0.14; *P* = .527). However, there was a positive correlation between the absolute difference in MAP and the dogs' body weight (Pearson correlation coefficient: 0.69; *P* < .001).

### Measurements at the pelvic limb

Details of ABP values measured at the pelvic limb are presented in Table 2. For SAP (*P* = .030), MAP (*P* = .015) and DAP (*P* = .025), values were significantly decreased when the limb was elevated with respect to the heart base (Fig 3). The absolute difference in SAP values measured at heart base and below was 5.9 (0.9 to 10.9) mmHg. There was no significant correlation between the magnitude of that difference and either the age of the dogs (Pearson correlation coefficient: 0.25; *P* = .269) and their body weight (Pearson correlation coefficient: 0.31; *P* = .163).

**Table 2:** Values of systolic (SAP), mean (MAP) and diastolic (DAP) arterial blood pressure measured in 22 anaesthetised dogs at the pelvic limb at the level of the heart base, and above and below the heart base

Measurements	Medians (ranges)	Means ± SD	Test	*P* value
**SAP (mmHg)**				
Heart base	120 (103 to 137)	121 ± 17		
Above heart base	117 (100 to 134)	117 ± 17	Paired *t*‐test^‡^	.030*
Below heart base	122 (103 to 141)	122 ± 19	Paired *t*‐test^‡^	.374
**MAP (mmHg)**				
Heart base	85 (66 to 102)	85 ± 17		
Above heart base	82 (64 to 95)	82 ± 18	Paired *t*‐test^‡^	.015*
Below heart base	87 (80 to 104)	87 ± 17	Paired *t*‐test^‡^	.395
**DAP (mmHg)**				
Heart base	65 ± 19	65 (46 to 80)		
Above heart base	61 ± 19	61 (42 to 80)	Paired *t*‐test^‡^	.025*
Below heart base	66 ± 18	66 (48 to 84)	Paired *t*‐test^‡^	.529

^‡^ For comparison with baseline values

* *P* values are statistically significant

**FIG 3:**
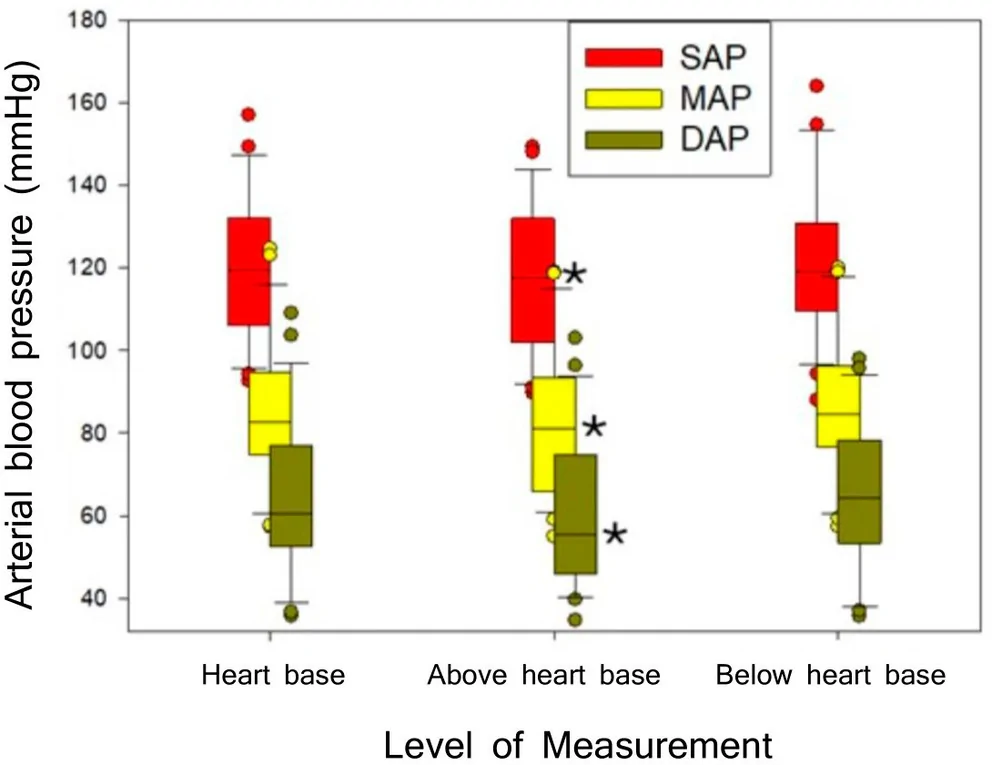
Values of systolic (SAP), mean (MAP) and diastolic (DAP) arterial blood pressures measured with PetMAP Graphic II at the pelvic limb of 22 anaesthetised dogs, with the cuff positioned at the level of the heart (baseline) and above and below the heart. The boxes represent the second and third quartiles, the horizontal line inside the boxes the medians and the vertical lines either side of each box the lower (25%) and upper (75%) quartiles. The dots represent the outliers. The stars indicate statistical significance.

The absolute difference in MAP values measured at heart base and above was 6.1 (1.6 to 10.6) mmHg. There were no significant correlations between that difference and either the age of the dogs (Pearson correlation coefficient: 0.13; *P* = .557) or the body weight of the dogs (Pearson correlation coefficient: 0.13; *P* = .145).

The absolute difference in DAP values measured at the heart base and above was 6.2 (1.5 to 10.9) mmHg. There was no significant correlation between that difference and either the age of the dogs (Pearson correlation coefficient: 0.40; *P* = .066) or their body weight (Pearson correlation coefficient: 0.30; *P* = .168).

## DISCUSSION

This study showed that, for ABP values measured at the thoracic limb, both SAP and MAP increase when the limb is lowered with respect to the heart base, with heavier dogs showing changes in MAP at greater magnitude. For measurements taken at the pelvic limb, SAP, MAP and DAP decrease when the limb is elevated.

Although these findings were expected based on previously published literature and clinical experience, they were not consistent between limbs. As a possible explanation for this, it is reasonable to hypothesise that deviations greater than 3 cm would have resulted in more consistent measurements across limbs. The distance measuring cuff‐heart base was calculated based on the assumption that the vertical depth of the thorax would represent a valid parameter to obtain comparable values across dogs of different sizes and breeds. In retrospect, in the absence of an identifiable more valid reference measure, standardisation of either breeds or canine morphotype and size could have strengthened the study methodology. Nevertheless, this approach would have complicated recruitment and limited the applicability of the findings to a single breed, whereas the aim was to generalise them across the canine population. In addition to this, confounding variables such as body condition score, shape of the chest and length of the limbs – which can vary between individual dogs even within the same breed – would not have been accounted for.

One question that remains unanswered is why body weight only affected the set of measurements taken at the thoracic limbs when the latter were lowered. It is possible that, when lowering the limbs, the effect of gravity and weight would add synergistically to that produced by the distance from the heart base to ultimately increase the magnitude of the change in ABP reading. Similar physical effects would not be expected when the limbs were elevated, also because they were physically supported. However, it remains unclear why an effect of body weight was only detected when the thoracic, but not the pelvic limbs, were lowered. Similar to the current study, a previous study conducted in cats also found that changes in ABP readings when lowering or elevating the limbs were not consistent across sets of measurements (Franchino et al., 2024).

The clinical implication of the study findings is that even small deviations of the cuff position, as little as 3 cm above or below the heart base, can result in considerable SAP and MAP discrepancies which, for the pelvic limb, can be as high as 6 mmHg. This magnitude of discrepancy may represent a clinically significant threshold, affecting the anaesthesiologists' decisions and actions resulting in either unnecessary and potentially detrimental administration of fluids and vasopressors, or omission of necessary interventions. This further emphasises the importance of ensuring that reliable ABP measurements are obtained with both the cuff and limb correctly positioned prior to confirming states of hypo‐ or hypertension and act accordingly. In addition to this, the Association for the Advancement of Medical Instrumentation (2028) considers differences in subsequent measurements of SAP below 5 mmHg as an acceptable error during NIBP monitoring in human patients. Although there are no recognised standards in veterinary patients, it is reasonable to suggest that reading errors above 5 mmHg may be clinically relevant.

Despite efforts to prevent or reduce measurement pitfalls, it is important to emphasise that previous studies on reliability of oscillometry indicate that many devices are most reliable within ABP values within normal ranges, with accuracy declining under conditions of marked hypertension or hypotension. (Marynissen et al., 2023; Sedgwick et al., 2021). Particularly the PetMAP seems to underestimate SAP at high blood pressure values (Walter et al., 2025). Therefore, the magnitude of under‐ and overestimation of ABP values when the limb position deviates from standard may be very different in hemodynamically unstable dogs compared to healthy dogs undergoing elective procedure, as it was the case in the current study.

This study has limitations. The findings are only applicable to healthy, anaesthetised dogs. The anaesthetic protocol was not standardised, and varying doses of alpha‐2 adrenoreceptor agonists may have produced cardiovascular effects of differing magnitudes, potentially influencing ABP measurements. However, because the focus of this study was on detecting ABP changes following limb repositioning rather than absolute ABP values, and since each dog served as its own control, it is unlikely that drug‐induced cardiovascular effects substantially affected the results. Other possible limitations are the lack of breed standardisation, and the fact that the sample size was determined based on estimations and assumptions, including the relationship between canine and human ventricle size when designing the equation, and the validity of the vertical depth of the thorax as a parameter to determine the deviation from the heart base. While the absence of invasive ABP measurements in the study dogs may be considered a limitation, it is important to note that validating the absolute accuracy of the PetMap against the invasive method was not the objective of this study and was previously addressed by former investigations (Vachon et al., 2014; Walter et al., 2025). Rather, the study was designed to address a different research question: to evaluate the effect of limb position relative to heart base level on ABP measurements obtained using the device.

In conclusion, in healthy anaesthetised dogs, ABP values measured with the PetMAP II, particularly SAP and MAP, tended to be underestimated when the limb was positioned above the level of the heart base and overestimated when positioned below it. For measurements taken at the pelvic limb, even small deviations as little as 3 cm above or below the heart base level resulted in mean SAP and MAP misreadings up to 6 mmHg, representing a potentially clinically relevant discrepancy.

## Author contributions

**R. McConomy:** Conceptualisation, data collection, methodology, resources, project administration and writing—original draft. **H. Pinnok:** Recruitment and data collection, and writing—review and editing. **C. Adami:** Conceptualisation, formal analysis, methodology, resources, software, supervision, visualisation, writing—original draft, and writing—review and editing.

## Conflict of interest

Prof. Adami is an associate editor of the *Journal of Small Animal Practice* and co‐author of this article. She was excluded from editorial decision‐making related to the acceptance of this article for publication. No other conflicts of interest have been declared.

## Funding information

BeneView T5, Mindray Bio‐Medical Electronics Ltd., China. Sedator, Dechra, United Kingdom. Dexdomitor, Espoo, Finland. Methadyne, Zoetis, United Kingdom. Propoflo, Zoetis, United Kingdom. Lidocaine hydrochloride 2%, Nexus, United Kingdom. Rimadyl, Zoetis, United Kingdom. Vetergesic, Ceva, United Kingdom. IBM SPSS Amos v 29, IL, USA, and SigmaStat version 4.0 & SigmaPlot version 10.0, CA, USA.

## AI disclosure statement

No generative artificial intelligence (AI) tools were used to generate, write or revise any part of this article.

## Data Availability

The original contributions presented in this study are included in this article; further inquiries can be directed to the corresponding author.

## References

[jsap70148-bib-0001] Acierno, M.J. , Brown, S. , Coleman, A.E. , Jepson, R.E. , Papich, M. , Stepien, R.L. et al. (2018) ACVIM consensus statement: guidelines for the identification, evaluation, and management of systemic hypertension in dogs and cats. Journal of Veterinary Internal Medicine, 32, 1803–1822. Available from: 10.1111/jvim.15331 PMC6271319 30353952

[jsap70148-bib-0002] Armour, M. , Michou, J. , Schofield, I. & Borland, K. (2024) Agreement of Doppler ultrasound and visual sphygmomanometer needle oscillation with invasive blood pressure in anaesthetised dogs. Animals (Basel), 14, 2756. Available from: 10.3390/ani14192756 PMC11475731 39409705

[jsap70148-bib-0003] Association for the Advancement of Medical Instrumentation (AAMI) . (2028) ANSI/AAMI/ISO 81060‐2:2018. Non‐invasive sphygmomanometers: part 2: clinical investigation of intermittent automated measurement type. Available from: https://www.iso.org/standard [Accessed 29th April 2026].

[jsap70148-bib-0004] Bailey, K. , Briley, J. , Duffee, L. , Duke‐Novakovski, T. , Grubb, T. , Kruse‐Elliott, K. et al. (2025) The American College of Veterinary Anesthesia and Analgesia Small Animal Anesthesia and Sedation Monitoring Guidelines. Veterinary Anaesthesia and Analgesia, 52, 377–385. Available from: 10.1016/j.vaa.2025.03.015 40447502

[jsap70148-bib-0005] Byrd, J.B. & Brook, R.D. (2014) Arm position during ambulatory blood pressure monitoring: a review of the evidence and clinical guidelines. Journal of Clinical Hypertension (Greenwich), 16, 225–230. Available from: 10.1111/jch.12255 PMC8032042 24467382

[jsap70148-bib-0006] Crippa, L. , Ferro, E. , Melloni, E. , Brambilla, P. & Cavalletti, E. (1992) Echocardiographic parameters and indices in the normal beagle dog. Laboratory Animals, 26, 190–195. 10.1258/002367792780740512 1501432

[jsap70148-bib-0007] de Almeida Lupiano, H.E. , Hofmeister, E.H. & Liao, P.T. (2025) Intra‐rater reliability, expertise and justifications for the American society of anesthesiologists physical status classification in dogs. Veterinary Anaesthesia and Analgesia, 52, 747–754. 10.1016/j.vaa.2025.09.003 41038722

[jsap70148-bib-0008] Franchino, G. , Fordyce, P. & Adami, C. (2024) Effect of limb and cuff positioning on measurement of arterial blood pressure with an oscillometry device (PetMAP) in anaesthetized cats. The Veterinary Journal, 305, 106137. Available from: 10.1016/j.tvjl.2024.106137 38759724

[jsap70148-bib-0009] Haahr, M. (2026) RANDOM.ORG: True Random Number Service. Available from: https://www.random.org [Accessed 1st February 2026].

[jsap70148-bib-0010] IMAIOS Vet‐Anatomy Atlas . (2026) Available from: https://www.imaios.com/en/vet‐anatomy/dog/dog‐general‐anatomy [Accessed 1st February 2026].

[jsap70148-bib-0011] Lyberg, M. , Ljungvall, I. , Häggström, J. , Ahlund, E. & Pelander, L. (2021) Impact of equipment and handling on systolic blood pressure measurements in conscious dogs in an animal hospital environment. Journal of Veterinary Internal Medicine, 35, 739–746. Available from: 10.1111/jvim.16062 PMC7995364 33586197

[jsap70148-bib-0012] Mantovani, M.M. , Costa, A.C.A. , de Lima, M.T. , Santos, L.F.N.D. , Silva, K.F. , Martins, A.R.C. et al. (2025) Agreement and diagnostic accuracy of new linear deflection oscillometry and doppler devices for hypotension detection compared to invasive blood pressure in anesthetized dogs. Veterinary Sciences, 12, 116. Available from: 10.3390/vetsci12020116 PMC11860341 40005875

[jsap70148-bib-0013] Marynissen, S. , Schils, G. , Stammeleer, L. , Daminet, S. , Smets, P. & Paepe, D. (2023) Systolic blood pressure measurements with Doppler ultrasonic flow detector and high‐definition oscillometry are comparable on population level but show large intra‐individual differences in apparently healthy elderly dogs. Journal of the American Veterinary Medical Association, 261, 1–8. 10.2460/javma.22.12.0579 36853876

[jsap70148-bib-0015] Rattez, E.P. , Reynolds, B.S. , Concordet, D. , Layssol‐Lamour, C.J. , Segalen, M.M. , Chetboul, V. et al. (2010) Within‐day and between‐day variability of blood pressure measurement in healthy conscious beagle dogs using a new oscillometric device. Journal of Veterinary Cardiology, 12, 35–40. Available from: 10.1016/j.jvc.2009.09.003 20185378

[jsap70148-bib-0016] Sedgwick, S. , Lorenzutti, A.M. , Araos, J.B. , Gleed, R.D. & Martin‐Flores, M. (2021) Evaluation of an oscillometric blood pressure monitor in anesthetized dogs: agreement with direct measurements and ability to detect hypotension. Research in Veterinary Science, 135, 162–166. Available from: 10.1016/j.rvsc.2021.01.016 33524826

[jsap70148-bib-0018] Vachon, C. , Belanger, M.C. & Burns, P.M. (2014) Evaluation of oscillometric and Doppler ultrasonic devices for blood pressure measurements in anesthetized and conscious dogs. Research in Veterinary Science, 97, 111–117. Available from: 10.1016/j.rvsc.2014.05.003 24924217

[jsap70148-bib-0019] Vet Visions . (2026) Small animal cardiology: auscultation. Available from: https://www.vetvisions.com/small‐animal‐cardiology‐auscultation/ [Accessed 15th January 2026].

[jsap70148-bib-0020] Walter, H. , Kästner, S.B.R. , Amon, T. & Tünsmeyer, J.M.A. (2025) Evaluation of two veterinary oscillometric noninvasive blood pressure (NIBP) measurement devices (petMAP Graphic II and high‐definition oscillometry) in dogs. Veterinary Sciences, 12, 349. Available from: 10.3390/vetsci12040349 PMC12031224 40284851

